# *Candida* periprosthetic joint infections — risk factors and outcome between *albicans* and non-*albicans* strains

**DOI:** 10.1007/s00264-021-05214-y

**Published:** 2021-11-16

**Authors:** Daniel Karczewski, Yi Ren, Octavian Andronic, Doruk Akgün, Carsten Perka, Michael Müller, Arne Kienzle

**Affiliations:** 1 Center for Musculoskeletal Surgery, Clinic for Orthopedics, Charité University Hospital, Charitéplatz 1, 10117 Berlin, Germany; 2 Department of Orthopaedics, Balgrist University Hospital, University of Zurich, Forchstrasse 340, 8008 Zurich, Switzerland; 3 Berlin Institute of Health at Charité – Universitätsmedizin Berlin, BIH Biomedical Innovation Academy, Charitéplatz 1, 10117, Berlin, Germany

**Keywords:** Periprosthetic joint infection, *Candida*, Fungal infection, Revision arthroplasty, Total knee arthroplasty, Total hip arthroplasty

## Abstract

**Background:**

Despite its scarcity, fungal periprosthetic joint infection (PJI) is of great clinical relevance as diagnosis and treatment are highly challenging. Previous analyses focused on the treatment rather than the role of the causative fungal agent on clinical outcome. This is the largest study of its kind to evaluate *Candida* strain–dependent differences in patients with fungal PJI.

**Methods:**

We retrospectively analyzed 29 patients who underwent surgical intervention due to *Candida* hip or knee PJI in our department from 2010 to 2018. PJI was defined according to IDSA, recurrent PJI according to modified Delphi consensus criteria. Statistical analysis was performed using *t*-test, chi-square test with Yates correction, and log rank test.

**Results:**

Besides age and affected joint, no significant differences were found between *Candida albicans* and non-*albicans* PJI patients (75.83 versus 64.11 years, *p* = 0.012; 12 hip versus two knee cases, *p* = 0.013). Most patients received two- (27.59%) or three-stage exchange surgery (41.38%). There was a statistical trend towards an increase in surgery needed in non-*albicans Candida* PJI (2.92 versus 2.12; *p* = 0.103). After initial *Candida* PJI treatment, functional prosthesis implantation was achieved in 72.41% of all patients. At last follow-up, infection-free survival was at 26.79% in *Candida albicans* versus 72.00% in non-*albicans* PJI (*p* = 0.046).

**Conclusions:**

In this study, we found infection-free survival rates to be significantly decreased in patients with *albicans* compared to non-*albicans Candida* PJI. While age and affected joint might play a confounding role, we speculate the causative pathogen to play a decisive role in disease progression.

**Supplementary Information:**

The online version contains supplementary material available at 10.1007/s00264-021-05214-y.

## Introduction

Despite increased routine usage of antibiotic prophylaxis and improved aseptic surgical techniques, periprosthetic joint infection (PJI) is still a common yet severe complication after both TKA and THA [1–5]. While the vast majority of cases are made up by bacterial infections, in approximately 1% of all cases, PJI is due to fungal microorganisms [6, 7]. In spite of its scarcity, detailed understanding of fungal PJI is of great clinical relevance as diagnosis and treatment is highly challenging for physicians and patients alike [8]. Additionally, treatment of fungal PJI is usually complicated by additional severe diseases impacting the immunocompetence in these affected patients [9, 10]. While an interdisciplinary team of surgeons, microbiologists, and infectious disease specialists is considered optimal, patients often undergo several procedures until being diagnosed and treated accordingly at a specialized centre [11, 12]. As there are no biofilm-active antimycotics available, surgical intervention and antifungal therapy are obligatory to achieve a successful, infection-free outcome [13, 14]. Revision surgery typically comprises removal of the infected prosthesis, debridement, irrigation, and introduction of a temporary arthrodesis followed by reimplantation of a new prosthesis in a second-stage surgery [15]. While there is broad consensus regarding treatment of bacterial PJI, there is paucity of data to validate an agreed upon treatment protocol for fungal PJI [16]. Besides prosthesis exchange surgery, single case reports have suggested implant retention or permanent resection arthroplasty of the hip and arthrodesis of the knee joint to be viable treatment options [17–22].

A range of case reports and smaller case series have investigated patient demographics and outcomes in patients suffering from fungal PJI [20, 23, 24]. However, previous systematic analyses have focused on the impact of treatment rather than the role of the causative fungal agent on clinical outcome [9, 10]. Fungal PJI comprises a range of different microorganisms. Commonly, fungal PJI is due to various *Aspergillus* or *Candida* strains [25]. While in most cases fungal PJI is caused by *Candida* [6, 18], relative prevalence of the different *Candida* strains in PJI is unknown. Besides *Candida albicans*, cases of *Candida freyschussii*, *Candida glabrata*, *Candida tropicalis*, and *Candida parapsilosis* PJI have been previously described [26–30]. However, despite its clinical relevance, little research has focused on systematically exploring differences in distribution, clinical features, and outcome. This lack of knowledge on *Candida* strain–dependent patient characteristics and clinical outcome leads to a paucity in differential and targeted treatment approaches.

In this study, we retrospectively analyzed the cases of 29 patients who underwent surgical intervention due to *Candida* PJI. This is the largest study of its kind to evaluate *Candida* strain–dependent differences in patients affected by this pathology. Patient demographics, risk factors, clinical and paraclinical parameters, and long-term outcome were assessed and compared for patients suffering from *Candida albicans* PJI and non-*albicans Candida* PJI.

## Material and methods

### Patients

This study was approved by the institutional ethics board and completed in accordance with the Declaration of Helsinki. We retrospectively studied all patients that were diagnosed with PJI with a *Candida* strain and subsequently underwent PJI-dependent TKA or THA revision surgery in between 2010 and 2018 at our hospital. A total of 29 patients were included in this study. Exclusion criterion was the following: diagnosed PJI lacking positive microbiological culture for any *Candida* strain. There were no further exclusions. Clinical and paraclinical parameters were evaluated for all patients. PJI was defined according to IDSA criteria as previously described [31]. For all statistical assessments in this study, patients were divided into the two groups depending on the detected *Candida* strain: *Candida albicans* PJI and non-*albicans Candida* PJI patients.

### Treatment

Patients received centralized and interdisciplinary treatment in our specialized department for total joint replacement and infections. Antibacterial and antifungal therapies were initiated as soon as PJI was diagnosed and was continuously adapted according to found microbial susceptibilities and resistances. Antimicrobial treatment was chosen based on bacterial susceptibility, the recommendations of Zimmerli [32], and in consultation with our department for microbiology and infectiology.

For revision surgery, one-, two-, or multiple-stage exchange surgery was performed as previously described [15, 33, 34]. After removal of the infected prosthesis, patients received a temporary, antibiotic-loaded cement spacer between stages in case of two- or multiple-stage exchange surgery. Antibiotics were chosen based on microbial susceptibilities and resistances or empirically. Prosthesis reimplantation was performed at least six weeks after removal with no clinical and laboratory signs of infection apparent. In few cases, permanent girdlestone resection arthroplasty or DAIR was performed at patients’ behest or if reimplantation was not possible due to paraclinical parameters. All surgical procedures were conducted by high-volume surgeons specialized in the treatment of PJI. Postoperatively, all patients received long-term antifungal suppression treatment through our outpatient department or were advised to continue treatment through their local orthopaedic doctor.

### Microbiology

Blood samples, joint aspirate, and intra-operative specimens were assessed by our clinical medicine and microbiology department. Patients were grouped depending on the detected fungal pathogen into *Candida albicans* and non-*albicans Candida* PJI patients. Pathogens found in latter group consisted of *Candida tropicalis*, *Candida parapsilosis*, and *Candida guilliermondii*. Mixed fungal infections with both *Candida albicans* and non-*albicans Candida* were diagnosed in two cases and were assigned to the *Candida albicans* group.

### Follow-up

All patients were regularly invited to our specialized outpatient clinic for follow-up examinations. Patients were invited six to 12 weeks post-operatively and thereafter for annual follow-ups. Patients that could not visit our outpatient department due to severe comorbidities were invited to follow-ups by phone. Complications of revision arthroplasty comprise, among others, recurrent PJI with the same or a different pathogen, aseptic loosening, instability, pain, patellar maltracking, arthrofibrosis, and limited range of motion. For this study, endpoints were defined as recurrent PJI, revision surgery-, or PJI-associated death. Recurrent PJI was diagnosed using IDSA and modified Delphi criteria [31, 35].

### Statistics

All data were analyzed using GraphPad Prism 7 software (GraphPad Software, La Jolla, CA, USA) and Excel (v16.30; Microsoft Corporation, Redmond, WS, USA). All data are presented as mean ± one standard deviation. Statistical analysis was performed using one-way analysis of variance. Unpaired Student’s *t*-test for samples of unequal variances, and chi-squared test with Yates correction was used to test for statistical significance (*p* < 0.05). Where applicable, *p* values are listed.

## Results

### Patient characteristics

Patient characteristics are outlined in Table 1. Of the 29 patients included in our analysis, 13 were male and 16 were female. Average patient age was 70.98 ± 12.72 years. Mean duration of follow-up after the last surgical intervention was 33.03 months (range, 0.37 to 119.13 months). Affected joints were the hip in 14 patients and knee in 15 patients. Mean Charlson Comorbidity Index (CCI) was 5.38 (range, 2 to 10). Most patients had an ASA score of 2 (13.79%) or 3 (82.76%).

**Table 1:** Patient characteristics

Descriptive	All patients	*Candia albicans* PJI	Non-*albicans Candida* PJI	*p* value
Count [number]	29	17	12	-
Gender distribution [count]	13 males; 16 females	5 males; 12 females	8 males; 4 females	0.108
Age [years]	70.98 ± 12.72	75.83 ± 7.29	64.11 ± 15.71	0.012
Follow-up time [months]	33.03 ± 32.80	26.70 ± 33.48	42.01 ± 30.95	0.223
Affected joint [count]	Hip: 14; knee: 15	Hip: 12; knee: 5	Hip: 2; knee: 10	0.013
*Clinical scores*				
CCI	5.38 ± 2.09	5.88 ± 2.00	4.67 ± 2.10	0.126
ASA1 [%] (number of patients [count])	3.45% (1)	0.00% (0)	8.33% (1)	-
ASA2 [%] (number of patients [count])	13.79% (4)	5.88% (1)	25.00% (3)	-
ASA3 [%] (number of patients [count])	82.76% (24)	94.12% (16)	66.67% (8)	-

For all statistical analyses, patients were grouped into patients suffering from *Candida albicans* PJI versus non-*albicans Candida* PJI. Patients in the *Candida albicans* group were significantly older (+ 11.72 years; *p* = 0.012) compared to the non-*albicans Candida* patients. *Candida albicans* PJI affected the hip joint significantly more often, while in the non-*albicans Candida* group, the knee joint was most affected. No significant differences for any of the other analyzed parameters were found.

### Diagnosis and treatment of PJI

Pathologically elevated CRP (> 5 mg/L) was found in all but three patients pre-operatively (Table 2). Mean CRP was 51.74 mg/L (range, 3.70 to 249.40 mg/L). Pre-operative prevalence of leukocytes was at physiological levels (normal range 3.90 to 10.50 cells/nL) in all but four patients that showed leukocytosis. No significant differences in CRP (range, 4 to 249 mg/L) and leukocytes (range, 11.10 to 26.10 cells/nL) were found in between *Candida albicans* PJI and non-*albicans Candida* PJI patients (*p* = 0.111 and *p* = 0.133). Polymicrobial infection with additional bacterial pathogens was found in 75.86% of all cases (76.47% for *Candida albicans* PJI and 75.00% for non-*albicans Candida* PJI).

**Table 2:** Laboratory analysis of bloodwork and microbiology

Descriptive	All patients	*Candia albicans* PJI	Non-*albicans Candida* PJI	*p* value
*Laboratory analysis*				
CRP [mg/L]	51.74 ± 60.57	66.85 ± 72.53	30.33 ± 28.88	0.111
Leukocytes [cells/nL]	8.96 ± 4.92	10.12 ± 6.04	7.32 ± 1.88	0.133
*Microbiology*				
*Candida* strain [%] (Number of Patients [count])				
~ *albicans*	58.62% (17)	100.00% (17)	0.00% (0)	-
~ *glabrata*	3.45% (1)	5.88% (1)	0.00% (0)	-
~ *parapsilosis*	34.48% (10)	0.00% (0)	83.33% (10)	-
~ *guilliermondii*	3.45% (1)	0.00% (0)	8.33% (1)	-
~ *tropicalis*	3.45% (1)	0.00% (0)	8.33% (1)	-
Additional bacterial infection [%] (Number of Patients [count])	75.86% (22)	76.47% (13)	75.00% (9)	-

A substantial number of patients had received revision arthroplasty due to aseptic loosening (16 patients), non-fungal PJI (14 patients), or — at independent time points — both (1 patient) prior to suffering from *Candida* PJI (Table 3). In the majority of patients (12 in the *Candida albicans* group and nine in the non-*albicans Candida* group), bacterial PJI was diagnosed prior to the *Candida* PJI diagnosis (mean, 258.10 days; range, 3 to 1273 days). No significant differences in mean time between diagnosis of bacterial and *Candida* PJI was found for the *Candida albicans* (mean, 204.75 days) and non-*albicans Candida* group (mean, 329.22; *p* = 0.183). In five cases, *Candida* PJI was the first diagnosed PJI and, in three cases, time between bacterial and *Candida* PJI was unknown.

**Table 3:** Previous surgeries and PJI treatment

Descriptive	All Patients	*Candia albicans* PJI	non-*albicans Candida* PJI	*p* value
*Prior Prosthesis Exchange Surgeries*				
…due to non-*Candida* PJI [%] (Number of Patients [count])	48.28% (14)	52.94% (9)	41.67% (5)	-
…due to Aseptic Loosening [%] (Number of Patients [count])	55.17% (16)	64.71% (11)	41.67% (5)	-
*Surgical treatment*				
Number of surgical procedures [count]	2.45 ± 1.30	2.12 ± 1.32	2.92 ± 1.16	0.103
Duration of prosthesis-free Interval [months]	5.75 ± 5.81	5.33 ± 6.82	6.12 ± 5.09	0.776
Negative pressure wound therapy [%] (number of patients [count])	37.93% (11)	41.18% (7)	33.33% (4)	-
*Antifungal treatment*				
…with fluconazole	86.21% (25)	82.35% (14)	91.67% (11)	-
…with caspofungin	13.79% (4)	17.65% (3)	8.33% (1)	-
…with voriconazole	6.90% (2)	11.76% (2)	0.00% (0)	-
Additional antibiotics treatment [%] (number of patients [count])	96.55% (28)	94.12% (16)	100.00% (12)	-

In average, patients received 2.45 surgical procedures and negative pressure wound therapy was administered in 37.93% of all cases. While the majority of patients received two- (27.59% of all patients) or three-stage exchange surgery (41.38% of all patients), two patients (6.90% of all patients) underwent one-stage exchange surgery or DAIR, respectively. In 17.24% of all cases, patients did not receive a new prosthesis after implant removal. There was a statistical trend towards an increase in surgery needed in patients with non-*albicans Candida* PJI (2.92 versus 2.12 in *Candida albicans* PJI; *p* = 0.103).

**Fig. 1:**
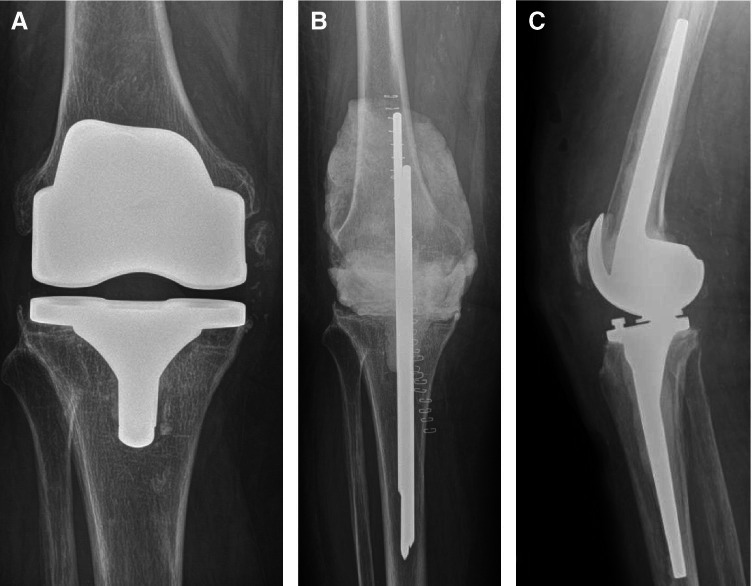
Representative patient who experienced *Candida parapsilosis* PJI after primary TKA and received two-stage exchange surgery

In case of two- or multiple-stage exchange surgery, mean duration of prosthesis-free interval was 5.75 months (range, 1.53 to 23.13 months) with no significant differences in between groups (*p* = 0.777). Figure 1 shows images of a representative patient who experienced *Candida parapsilosis* PJI after primary TKA and received two-stage exchange surgery.

The majority of patients (86.20%) received fluconazole alone or in combination with an additional antifungal agent. Caspofungin and voriconazole as sole antifungal therapy were administered to one patient each. At the patients’ behest, one patient did not receive antifungal or antibiotic treatment but palliative care instead. All other patients received antibiotic treatment. A minimum of 12 months of antifungal treatment was recommended to all patients at discharge. Individual microbiological results and treatment regimens are detailed in Supplemental Table 1.

### Outcome

After initial *Candida* PJI treatment, functional prosthesis implantation was achieved in 72.41% of all patients (Table 4). In eight patients, girdlestone resection arthroplasty of the hip or arthrodesis of the knee was the final outcome. PJI-associated death was reported for three patients at the last follow-up. No significant differences were found between groups at both time points.

**Table 4:** Outcome

Descriptive	All patients	*Candia albicans* PJI	Non-*albicans Candida* PJI	*p* value
*Outcome after Candida PJI treatment*				
Prosthesis (THA/TKA) [%] (number of patients [count])	72.41% (21)	70.59% (12)	75.00% (9)	0.793
Girdlestone resection arthroplasty/arthrodesis [%] (number of patients [count])	27.59% (8)	29.41% (5)	25.00% (3)	
*Outcome at final follow-up*				
Prosthesis (THA/TKA) [%] (number of patients [count])	62.07% (18)	52.94% (9)	75.00% (9)	0.367
Girdlestone resection arthroplasty/arthrodesis/amputation [%] (number of patients [count])	27.59% (8)	29.42% (5)	25.00% (3)	
PJI-associated death [%] (number of patients [count])	10.34% (3)	17.65% (3)	0.00% (0)	
Recurrent PJI [%] (number of patients [count])	27.59% (8)	35.29% (6)	16.67% (2)	-
Time to recurrent PJI (months)	10.37 ± 13.02	10.72 ± 14.98	9.32 ± 7.94	0.875

**Fig. 2:**
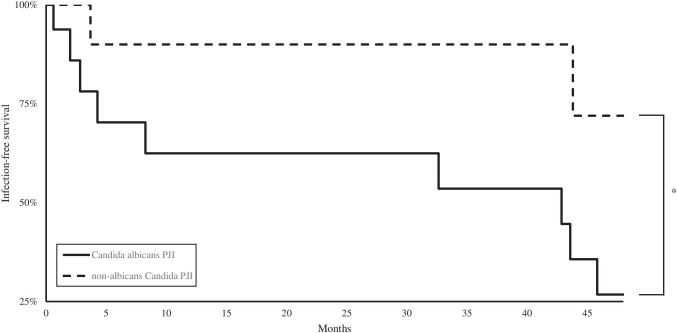
*Candida albicans* PJI and non-*albicans Candida* PJI

Infection-free survival was significantly lower in patients with *Candida albicans* PJI (Fig. 2; 26.79%) compared to patients with non-*albicans Candida* PJI (72.00%; *p* = 0.046). Recurrent PJI occurred in a total of eight patients after an average time of 10.37 months (range, 0.17 to 32.63 months): Recurrent PJI occurred in 35.29% of the patients in the *Candida albicans* and in 16.67% of the patients in the non-*albicans Candida* PJI group (Table 4).

## Discussion

In this study, we assessed clinical and paraclinical data to study potential differences in patient demographics, treatment, and clinical outcome in patients with PJI due to *Candida albicans* versus non-*albicans Candida* strains at our university hospital. Besides age and the affected joint, no significant differences were found in patient characteristics and treatment. However, long-term infection-free survival rate was significantly lower in patients with *Candida albicans* PJI compared to non-*albicans Candida* PJI.

In this study, we found *Candida albicans* followed by *Candida parapsilosis* to be the most commonly diagnosed *Candida* strains. While mean age was comparable to patients receiving primary TKA or THA [36], both ASA and CCI scores were higher in our patient collective suggesting comorbidities to be a significant risk factor. However, patients affected by *Candida albicans* PJI were significantly older compared to non-*albicans Candida* PJI patients. Of note, we found a substantial number of patients suffering from *Candida* PJI to have had prior revision arthroplasty due to aseptic loosening or non-fungal PJI. In accordance, with these results, previous reports highlighted an elevated risk for fungal PJI in older patients that had previously received revision arthroplasty [6]. Polymicrobial infections were found in approximately 75% of the cases in our study, compared to 19 to 36% in bacterial PJI [37, 38], suggesting impaired immune response in these patients.

While there is a strong tendency towards two- or multiple-stage revision surgery, reports on comparable outcomes after single-stage exchange surgery exist [21, 22]. However, due to its scarcity, there is no generally agreed upon treatment guidelines for fungal PJI. As this is the largest study of its kind to evaluate *Candida* PJI, previous reports have struggled to draw valid conclusions regarding differences in clinical features and outcome from smaller case series [16]. In most cases, treatment is further complicated by impacted immunocompetence in these affected patients [9, 10]. As evident in our patient collective, *Candida* PJI is in most cases only diagnosed after initial treatment for bacterial PJI leading to stark differences in surgical treatment. As there are no biofilm-active antimycotics available, re-revision and potentially an exchange to an antimycotic cement spacer might be necessary in such cases [13, 14]. However, if fungal PJI is diagnosed after prosthesis reimplantation, surgeons may opt to attempt to retain the implant with long-term antifungal therapy in severely diseased patients with a high intra-operative risk. With current diagnostic tools and guidelines, it may not be feasible to strictly follow an agreed-upon therapeutic approach in affected patients. This also limits the peri-operative use of antifungal medications and as an additive during cementation. In our experience, involved surgeons should consider fungal pathogens particularly if PJI persists despite adequate antibiotic and surgical treatment. Additionally, as seen in our patients, it may not be possible for severely diseased patients to receive a new implant after resection arthroplasty or arthrodesis due to soft-tissue constraints. Similarly, previous case reports have suggested permanent resection arthroplasty of the hip and arthrodesis of the knee joint as viable treatment options [17–20]. The lack of an agreed upon treatment protocol for fungal PJI and the resulting large differences in treatment in the literature and in our patient sample further complicates comparison of outcome [16]. Additionally, treatment regimens are commonly chosen depending on the surgeons’ experience and sometimes limited at the patients’ behest in these often highly complex cases. The lack of larger patient sample size warrants further research on optimal treatment of these patients.

While infection-free survival rates are at approximately 80 to 90%, risk of re-revision due to recurrent PJI or aseptic loosening is significantly elevated after primary bacterial PJI [33, 39]. Reviews on a number of published case reports and smaller case series have concluded treatment of fungal PJI to be markedly less successful with infection-free survival rates ranging from approximately 40 to 80% [7, 16, 17]. In our study, we found similar overall survival rates. However, clinical outcome was at only 27% in the *Candida albicans* PJI group. In contrast, infection-free survival in patients with non-*albicans Candida* PJI was at 72%. Additionally, we found a significantly greater number of patients with PJI of the hip in this group compared to in non-*albicans Candida* PJI patients. In accordance with these results, Brown et al. found patients with fungal PJI of the hip — independent of the fungal strain — to have significantly lower infection-free survival rates [6]. Previously, both ASA and CCI have been suggested to be useful in predicting risk of fungal PJI after revision arthroplasty [6, 40]. In contrast to these reports, clinical outcomes in both groups did not correlate to ASA or CCI in our patient collective.

Limitations of the current study include the heterogeneity of the analyzed population, the retrospective study design, and the analyzed cohort size with potential subsequent statistical bias.

In conclusion, we found infection-free survival rates to be significantly decreased in patients with *Candida albicans* PJI compared to non-*albicans Candida* PJI. While age and affected joint might play a confounding role, we speculate the causative pathogen to play a decisive role in disease progression.

## Supplementary Information

Below is the link to the electronic supplementary material.

Supplementary file1 (DOCX 35 kb)

Supplementary file2 (DOCX 26 kb)

## Abbreviations

CRP: C-reactive protein
PJI: Periprosthetic joint infection
TKA: Total knee arthroplasty
THA: Total hip arthroplasty
DAIR: Debridement, antibiotics, and implant retention
