# Supplementary material for: Candida periprosthetic joint infections — risk factors and outcome between albicans and non-albicans strains
Source: Int Orthop. 2021 Nov 16;46(3):449–56. doi: 10.1007/s00264-021-05214-y (PMC8840907; doi:10.1007/s00264-021-05214-y)
Supplement: Supplementary file 1 — Supplementary file1 (DOCX 35 kb) [file 264_2021_5214_MOESM1_ESM.docx]

**Supplemental Table 1.** Detailed Patient Characteristics and Microbiology Results

| **# Patient** | **Age** | **Sex** | **Affected Joint** | **Fungal Strain** | **Bacterial Infection** | **Antifungal Treatment** | **Surgical Treatment** | **Recurrent PJI** | **Time to Recurrent PJI** | **Long-Term Outcome** |
| --- | --- | --- | --- | --- | --- | --- | --- | --- | --- | --- |
| 1 | 80.94 | F | right hip | Candida albicans | Staphylococcus epidermidis | Fluconazole | One-stage exchange | No | - | THA |
| 2 | 79.70 | F | left hip | Candida albicans | Staphylococcus epidermidis | Caspofungin  Fluconazole | Three-stage exchange | No | - | Death |
| 3 | 56.99 | F | right hip | Candida albicans | Streptococcus mitis  Staphylococcus aureus  Klebsiella pneumoniae  Staphylococcus haemolyticus  Actinomyces neuii | Fluconazole | Two-stage exchange | Yes | 814 days | Girdlestone arthroplasty |
| 4 | 73.91 | M | left hip | Candida albicans | Staphylococcus epidermidis  Staphylococcus haemolyticus  Corynebacterium  Enterobacter cloacae | Fluconazole | Girdlestone resection arthroplasty | Yes | 85 days | Girdlestone arthroplasty |
| 5 | 63.95 | F | left knee | Candida albicans | - | Fluconazole | DAIR | Yes | 20 days | Knee arthrodesis |
| 6 | 81.93 | F | left knee | Candida albicans | - | Caspofungin | Three-stage exchange | Yes | 979 days | TKA |
| 7 | 75.08 | M | right hip | Candida albicans | Streptococcus aureus  Klebsiella oxytoca  Pseudomonas aeruginosa  Enterococcus faecalis | Fluconazole | Three-stage exchange | No | - | THA |
| 8 | 70.61 | F | right knee | Candida albicans | Enterococcus faecalis | Fluconazole | Two-stage exchange | No | - | TKA |
| 9 | 84.34 | F | right hip | Candida albicans | Pseudomonas aeruginosa | Fluconazole | Girdlestone resection arthroplasty | No | - | Girdlestone arthroplasty |
| 10 | 72.08 | F | left hip | Candida albicans | Staphylococcus aureus  Enterococcus faecium  Escherichia coli  Enterobacter cloacae  Providencia stuartii  Bacteroides uniformis  Staphylococcus epidermidis | Fluconazole | Girdlestone resection arthroplasty | Yes | 5 days | Girdlestone arthroplasty |
| 11 | 74.41 | M | left hip | Candida albicans | - | Fluconazole | Three-stage exchange | Yes | 26 days | THA |
| 12 | 80.82 | F | left hip | Candida albicans | Staphylococcus aureus  Staphylococcus epidermidis  Streptococcus mitis  Enterococcus faecium  Staphylococcus warneri | Fluconazole  Caspofungin | Two-stage exchange | No | - | THA |
| 13 | 79.01 | F | right knee | Candida albicans | Staphylococcus epidermidis | Fluconazole | DAIR | No | - | TKA |
| 14 | 82.05 | M | right hip | Candida albicans | Staphylococcus epidermidis  Klebsiella pneumoniae | Voriconazole | hemipelvectomy | No | - | Death |
| 15 | 71.89 | F | left knee | Candida albicans | - | Fluconazole | Two-stage exchange | No | - | TKA |
| 16 | 77.48 | F | left hip | Candida albicans  Aspergillus niger | Finegoldia magna | Fluconazole, Voriconazole | Three-stage exchange | No | - | THA |
| 17 | 83.86 | M | right hip | Candida albicans  Candida glabrata | Enterococcus faecium Citrobacter freundii  Enterococcus faecalis | none (palliative) | Girdlestone resection arthroplasty | No | - | Death |
| 18 | 60.62 | F | left knee | Candida tropicalis | Enterobacter cloacae  Citrobacter freundii  Streptococcus agalactiae  Enterococcus faecalis  Propionibacterium acnes  Streptococcus parasanguinis  Staphylococcus hominis  Bacillus subtilis  Staphylococcus lugdunensis  Staphylococcus epidermidis | Fluconazole | Three-stage exchange | Yes | 448 days | Amputation |
| 19 | 28.40 | M | left hip | Candida guilliermondii | Staphylococcus aureus  Cutibacterium acnes | unknown | Two-stage exchange | No | - | THA |
| 20 | 75.25 | F | right knee | Candida parapsilosis | - | Fluconazole | Three-stage exchange | No | - | Knee arthrodesis |
| 21 | 85.95 | F | right knee | Candida parapsilosis | - | Fluconazole | Two-stage exchange | No | - | TKA |
| 22 | 67.65 | M | left knee | Candida parapsilosis | Ralstonia pickettii | Fluconazole | One-stage exchange | Yes | 111 days | TKA |
| 23 | 76.00 | M | left knee | Candida parapsilosis | - | Fluconazole | Three-stage exchange | No | - | TKA |
| 24 | 59.95 | M | right hip | Candida parapsilosis | Bacillus cereus  Corynebacterium  Neisseria subflava  Streptococcus parasanguinis  Pseudomonas aeruginosa  Staphylococcus epidermidis | Fluconazole | Three-stage exchange | No | - | THA |
| 25 | 43.60 | M | left knee | Candida parapsilosis | Mycobacterium xenopi | Fluconazole | Three-stage exchange | No | - | Knee arthrodesis |
| 26 | 60.94 | M | right knee | Candida parapsilosis | Pseudomonas aeruginosa  Staphylococcus epidermidis  Enterococcus faecalis | Fluconazole | Three-stage exchange | No | - | TKA |
| 27 | 71.36 | F | right knee | Candida parapsilosis | Staphylococcus epidermidis  Propionibacterium avidum | Fluconazole  Caspofungin | Three-stage exchange | No | - | TKA |
| 28 | 62.41 | M | left knee | Candida parapsilosis | Staphylococcus lugdunensis | Fluconazole | Two-stage exchange | No | - | TKA |
| 29 | 77.15 | M | left knee | Candida parapsilosis | Staphylococcus epidermidis  Enterococcus faecalis | Fluconazole | Two-stage exchange | No | - | TKA |
