# Supplementary material for: Candida periprosthetic joint infections — risk factors and outcome between albicans and non-albicans strains
Source: Int Orthop. 2021 Nov 16;46(3):449–56. doi: 10.1007/s00264-021-05214-y (PMC8840907; doi:10.1007/s00264-021-05214-y)
Supplement: Supplementary file 2 — Supplementary file2 (DOCX 26 kb) [file 264_2021_5214_MOESM2_ESM.docx]

**Table 2.** Laboratory Analysis of Bloodwork and Microbiology

| **Descriptive** | **All Patients** | ***Candia albicans* PJI** | **non-*Candida albicans* PJI** | **p Value** |
| --- | --- | --- | --- | --- |
| *Laboratory Analysis* |  |  |  |  |
| CRP [mg/L] | 51.74±60.57 | 66.85±72.53 | 30.33±28.88 | 0.111 |
| Leukocytes [cells/nL] | 8.96±4.92 | 10.12±6.04 | 7.32±1.88 | 0.133 |
| *Microbiology* |  |  |  |  |
| Candida strain [%] (Number of Patients [count]) |  |  |  |  |
| ~ albicans | 58.62% (17) | 100.00% (17) | 0.00% (0) |  |
| ~ glabrata | 3.45% (1) | 5.88% (1) | 0.00% (0) |  |
| ~ parapsilosis | 34.48% (10) | 0.00% (0) | 83.33% (10) |  |
| ~ guillermondii | 3.45% (1) | 0.00% (0) | 8.33% (1) |  |
| ~ tropicalis | 3.45% (1) | 0.00% (0) | 8.33% (1) |  |
| Additional bacterial infection [%] (Number of Patients [count]) | 75.86% (22) | 76.47% (13) | 75.00% (9) |  |
